# Dataset on the 6-year radiocesium transport in rivers near Fukushima Daiichi nuclear power plant

**DOI:** 10.1038/s41597-020-00774-x

**Published:** 2020-12-15

**Authors:** Keisuke Taniguchi, Yuichi Onda, Hugh G. Smith, William Blake, Kazuya Yoshimura, Yosuke Yamashiki, Takayuki Kuramoto

**Affiliations:** 1 Center for Research in Isotopes and Environmental Dynamics, University of Tsukuba, Tsukuba, Ibaraki Japan; 2 Fukushima Prefectural Centre for Environmental Creation, Miharu, Fukushima Japan; 3 Landcare Research, Palmerston North, New Zealand; 4 School of Geography, Earth and Environmental Sciences, University of Plymouth, Plymouth, Devon UK; 5 Sector of Fukushima Research and Development, Japan Atomic Energy Agency, Minamisoma, Fukushima Japan; 6 Graduate School of Advanced Integrated Studies in Human Survivability, Kyoto University, Kyoto, Kyoto Japan; 7 School of Humanities and Culture, Tokai University, Hiratsuka, Kanagawa Japan

**Keywords:** Pollution remediation, Environmental monitoring

## Abstract

Radiocesium released from the Fukushima Daiichi nuclear power plant (FDNPP) and deposited in the terrestrial environment has been transported to the sea through rivers. To study the long-term effect of riverine transport on the remediation process near the FDNPP, a monitoring project was initiated by the University of Tsukuba. It was commissioned by the Ministry of Education, Culture, Sports, Science, and Technology, and the Nuclear Regulatory Commission in June 2011, and was taken over by the Fukushima Prefectural Centre for Environmental Creation from April 2015. The activity concentration and monthly flux of radiocesium in a suspended form were measured in the project. This provides valuable measurement data to evaluate the impact of the accidentally released radiocesium on residents and the marine environment. It can also be used as verification data in the development and testing of numerical models to predict future impacts.

## Abstract (summary)

**Table:** 

Measurement(s)	Cesium-137 Gamma Radiation • Cesium-134 Gamma Radiation • Latitude • Longitude • grain size • radiocesium flux • radioactivity concentration
Technology Type(s)	gamma-ray spectroscopy • GPS navigation system • laser diffraction particle size analyzer • Calculation
Factor Type(s)	geographic location
Sample Characteristic - Environment	suspended sediment
Sample Characteristic - Location	Abukuma River • Fukushima Prefecture • Miyagi Prefecture

Machine-accessible metadata file describing the reported data: 10.6084/m9.figshare.13055315

## Background & Summary

A 9.0 magnitude earthquake on March 11, 2011, caused the Tokyo Electric Power Company’s Fukushima Daiichi nuclear power plant (FDNPP) to be damaged by a tsunami, causing a large accident that spread radioactive materials into the environment^1,2^. This was the largest release of radioactivity into the environment since the Chernobyl nuclear power plant accident in 1986, and has been rated on the International Nuclear and Radiological Event Scale (INES) as a “Major Accident” by International Atomic Energy Agency (IAEA)^3^.

Radiocesium (^134^Cs and ^137^Cs) is a very important nuclide for evaluating the impact of the accident on the radiation risk in the medium- to long-term because of the significant release amount (10 PBq each) and the long-half-lives (2.07 and 30.1 years, respectively)^2^. Approximately 2.7 PBq of the total ^137^Cs release of 10 PBq was deposited on land in eastern Japan^4^.

In terrestrial environments, deposited radiocesium exists in particulate and dissolved forms. The former are those adsorbed on soil particles and the latter are dissolved in water as ions^5^. Although a part of the dissolved form existed as a colloidal form, the colloidal form ratio in the dissolved form was low^6^. During rainfall events, the surface soil that has absorbed radiocesium is eroded and transported into rivers, and finally reaches the ocean. Due to the high *K*_*d*_ values (i.e., the ratio between suspended and dissolved ^137^Cs concentrations)^7–12^, high levels of precipitation^13–15^, and steep topography^16^, >90% radiocesium was transported in the particulate form in Japanese rivers^7,8,10,17^. This ratio is much higher than in the rivers of Europe following the Chernobyl nuclear power plant (CNPP) accident^5,13^.

In response to the FDNPP accident, the Japanese government designated an evacuation zone based on the air dose rate (Transition of evacuation designated zones. *Fukushima Prefecture* https://www.pref.fukushima.lg.jp/site/portal-english/en03-08.html) and to conducted decontamination activities around the residential areas (Off-site Environmental Remediation in Affected Areas in Japan. *Ministry of the Environment* http://josen.env.go.jp/en/decontamination/). This resulted in regional differences in land-use conditions, with all human activities halted in the areas that received evacuation orders while agriculture in rice paddies and fields continued outside of these areas. The evacuation orders were lifted in stages as the decontamination process progressed. Continuous environmental monitoring is essential for safe habitation in these areas.

A long-term monitoring campaign began in June 2011, approximately three months after the accident, in order to comprehensively understand the movement of radiocesium in the terrestrial environment from the source to the ocean^18^. A considerable amount of data has been acquired and valuable knowledge has been provided, e.g., mapping of the air dose rate and initial deposition^19–21^, runoff from paddy fields^22^ and other land uses^23,24^, and riverine transport^10,17^. The riverine monitoring in the campaign was taken over by the Fukushima Prefectural Centre for Environmental Creation in April 2015 and is currently ongoing.

Data on radiocesium concentrations and fluxes collected in rivers within 80 km of the FDNPP from June 2011 (three months after the accident) to March 2017 (6 years after the accident) is available on the web pages^10,25–29^. The data is expected to be widely used for validation of the radiocesium transport model, comparison between the FDNPP and CNPP accidents, influence on the health of residents, and evaluation of the effect of the environmental remediation measures in Fukushima^30^. Additional data for Cs concentrations in size-fractionated sediments and information on size fractionation as a function of flow rate could be useful for further improving numerical models to predict future impacts.

## Methods

### Monitoring Sites

**Fig. 1:**
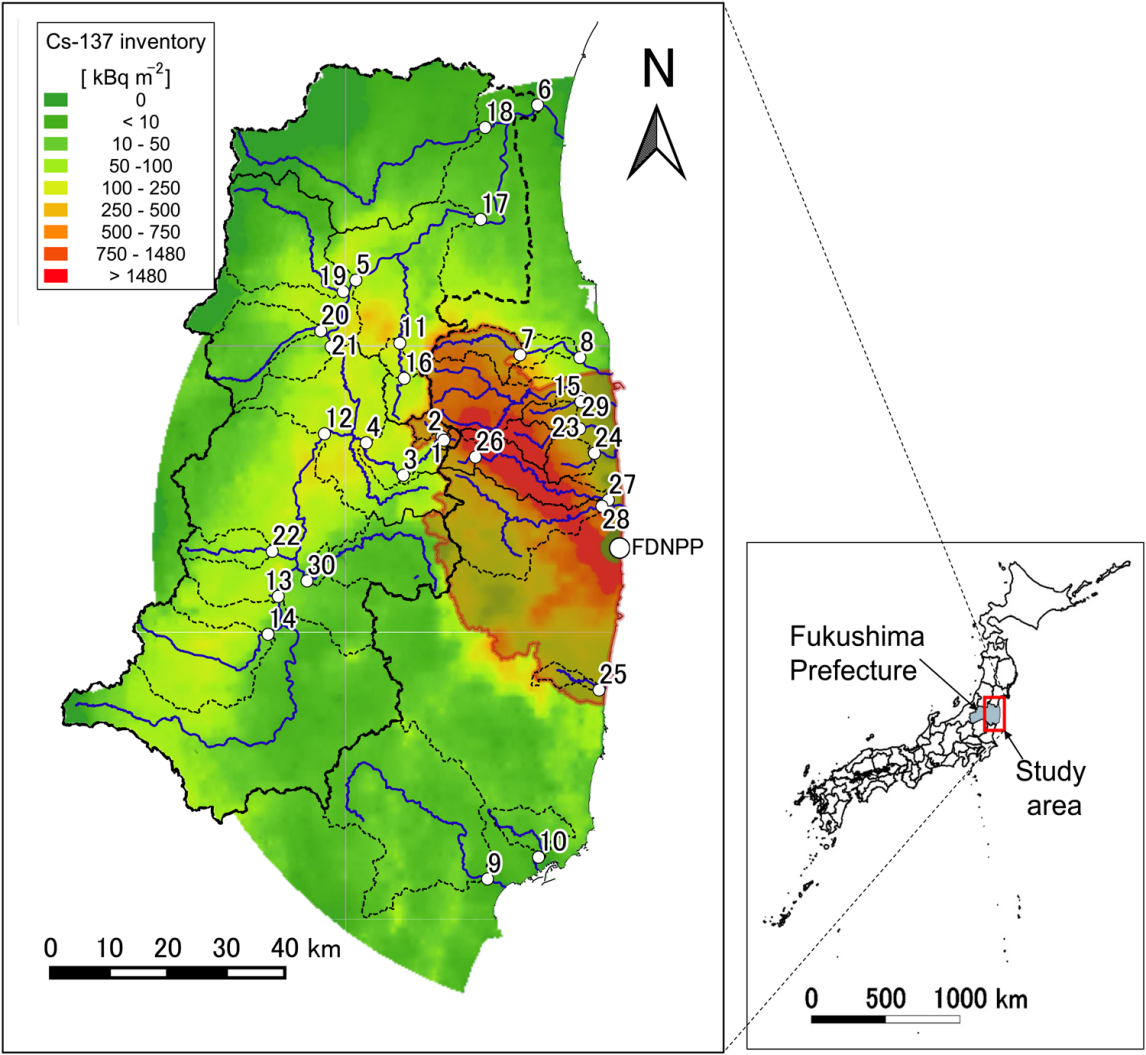
Monitoring site map. The numbers correspond to the site numbers in Table 1. The red shaded area is the area where the evacuation order was issued in the past. The solid blue line indicates the river channel where the observation points are located, and the area surrounded by black dotted lines indicates the catchment area of each observation point. The colour contours in the background of the map show the ^137^Cs deposition o as of July 2011^4^.

A total of 30 river observation sites were established within 80 km of the FDNPP (Fig. 1 and Table 1). Seventeen of these sites are located in the watershed of the Abukuma River which is the largest river system in the region. The remaining 13 sites are located in relatively small river systems on the coastal area of Fukushima Prefecture. The long-term monitoring sites (sites 1–6) and the other sites (sites 7–30) were respectively established in June 2011 and from October to December 2012 as a part of the monitoring campaigns by the Ministry of Education, Culture, Sports, Science and Technology (MEXT)^18^. These sites were selected because they were located in high ^137^Cs deposition areas and where data on water level and discharge could be obtained. The wide-spread monitoring network aimed to study temporal and regional variations in radiocesium transport through river networks to the ocean.

**Table 1:** Location of the observation points and period of data acquisition.

No.	Site name (abbreviation)	River name	River system	Latitude	longitude	*S*	*D*	Period of data availability	
						[km^2^]	[kBq/m^2^]	Radiocesium concentrations	Radiocesium flux
1	Mizusakai (Miz)	Kuchibuto	Abukuma	140°41′32″	37°35′08″	7.5	745.2	Jun.2011–Mar.2017	Jun.2011–Mar. 2017
2	Kuchibuto_Upper (KU)	Kuchibuto	Abukuma	140°41′18″	37°35′27″	21.4	477.4	Jun.2011–Mar.2017	Jun.2011–Mar. 2017
3	Kuchibuto_Middle (KM)	Kuchibuto	Abukuma	140°36′50″	37°31′55″	62.8	357.2	Jun.2011–Mar.2017	Jun.2011–Mar. 2017
4	Kuchibuto_Down (KD)	Kuchibuto	Abukuma	140°32′31″	37°34′52″	135.2	269.1	Jun.2011–Mar.2017	Jun.2011–Mar. 2017
5	Fushiguro (Fus)	Abukuma	Abukuma	140°31′12″	37°49′43″	3644.5	95.9	Jul.2011–Mar.2017	Jun.2011–Mar. 2017
6	Iwanuma (Iwa)	Abukuma	Abukuma	140°52′19″	38°05′41″	5313.2	88.4	Jul.2011–Feb.2017	Jun.2011–Mar. 2017
7	Mano (Man)	Mano	Mano	140°50′22″	37°42′57″	75.6	498.7	Aug.2011–Feb.2017	Oct.2012–Mar. 2017
8	Ojimadazeki (Oji)	Mano	Mano	140°57′19″	37°42′42″	110.8	405.5	Aug.2011–Feb.2017	Oct.2012–Mar. 2017
9	Matsubara (Mat)	Same	Same	140°46′43″	36°55′03″	570.9	40.0	Sep.2011–Feb.2017	Oct.2012–Mar. 2017
10	Onahama (Ona)	Fujiwara	Fujiwara	140°52′35″	36°57′03″	70.1	38.8	Sep.2011–Feb.2017	Oct.2012–Mar. 2017
11	Tsukidate (Tsuk)	Hirose	Abukuma	140°36′22″	37°43′59″	83.6	222.8	Aug.2011–Aug.2016	Oct.2012–Mar. 2017
12	Nihonmatsu (Nih)*	Abukuma	Abukuma	140°27′40″	37°35′40″	2380.3	81.8	Aug.2011–Mar.2017	Oct.2014*–Mar. 2017
13	Miyota (Miy)	Abukuma	Abukuma	140°22′25″	37°20′47″	1286.6	74.1	Sep.2011–Aug.2016	Oct.2012–Mar. 2017
14	Nishikawa (Nis)	Shakado	Abukuma	140°21′16″	37°17′19″	289.4	132.0	Sep.2011–Feb.2017	Oct.2012–Mar. 2017
15	Kitamachi (Kit)	Mizunashi	Niida	140°57“26″	37°38′40″	35.8	564.5	Aug.2011–Mar.2017	Oct.2012–Mar. 2017
16	Kawamata (Kaw)	Hirose	Abukuma	140°36′54″	37°40′46″	56.6	229.1	Aug.2011–Mar.2017	Oct.2012–Mar. 2017
17	Marumori (Mar)^*2^	Abukuma	Abukuma	140°45′44″	37°55′18″	4123.9	105.1	Dec.2012–Feb.2017	—
18	Funaoka-ohashi (Fun)^*3^	Shiroishi	Abukuma	140°46′14″	38°03′43″	775.2	20.2	—	—
19	Senoue (Sen)	Surigami	Abukuma	140°29′45″	37°48′40″	313.3	41.9	Dec.2012–Mar.2017	Oct.2012–Mar. 2017
20	Yagita (Yag)^*4^	Ara	Abukuma	140°27′10″	37°45′04″	184.6	52.7	Dec.2012–Apr.2016*^4^	Oct.2012–Mar. 2016*^4^
21	Kuroiwa (Kur)	Abukuma	Abukuma	140°28′23″	37°43′38″	2921.4	103.4	Dec.2012–Mar.2017	Oct.2012–Mar. 2017
22	Tomita (Tom)	Ouse	Abukuma	140°21′41″	37°24′54″	72.6	98.5	Dec.2012–Feb.2017	Oct.2012–Mar. 2017
23	Ota (Ota)	Ota	Ota	140°57′18″	37°36′12″	49.9	1767.5	Dec.2012–Mar.2017	Oct.2012–Mar. 2017
24	Odaka (Oda)	Odaka	Odaka	140°59′1″	37°33′58″	50.3	724.2	Dec.2012–Mar.2017	Oct.2012–Mar. 2017
25	Asami (Asa)	Asami	Asami	140°59′33″	37°12′20″	25.8	193.8	Dec.2012–Feb.2017	Oct.2012–Mar. 2017
26	Tsushima (Tsus)^*5^	Ukedo	Ukedo	140°45′12″	37°33′36″	25.4	951.5	Dec.2012–Mar.2017	Oct.2012–Aug. 2015*^5^
27	Ukedo (Uke)	Ukedo	Ukedo	141° 0′38″	37°29′41″	152.6	2565.9	Dec.2012–Mar.2017	Oct.2012–Mar. 2017
28	Takase (Tak)	Takase	Ukedo	140°59′53″	37°29′7″	263.7	726.0	Dec.2012–Mar.2017	Oct.2012–Mar. 2017
29	Haramachi (Har)	Niida	Niida	140°57′29″	37°39′4″	200.3	963.7	Dec.2012–Mar.2017	Oct.2012–Mar. 2017
30	Akanuma (Aka)	Otakine	Abukuma	140°25′44″	37°22′13″	242.6	52.6	Dec.2012–Mar.2017	Oct.2012–Mar. 2017

The geodetic datum for all locations is WGS1985 (ERSG = 4326).

*At point No. 12, fluxes could not be calculated due to the poor quality of turbidity data prior to April 2014

*2 At point No. 17, the flux could not be calculated due to the poor quality of turbidity data.

*3 There is no data on suspended radiocesium at point No. 18 because only water sampling was carried out at that point.

*4 Observations at No. 20 were suspended at the end of FY 2016 due to a major change in the shape of the flow channel caused by a typhoon.

*5 At point No. 26, due to the impact of the typhoon in September 2015, data from that point has not been released yet.

Following the FDNPP incident, evacuation orders have been issued for areas of concern for the health of residents (Transition of evacuation designated zones. *Fukushima Prefecture* https://www.pref.fukushima.lg.jp/site/portal-english/en03-08.html). The area covered a maximum of approximately 1600 km^2^ in 11 municipalities (Fig. 1). As of March 2020, the designated evacuation area has been reduced to approximately 330 km^2^ in seven municipalities (∼Fukushima Today∼Steps for Reconstruction and Revitalization in Fukushima Prefecture (2020.3.24). *Fukushima Prefecture* https://www.pref.fukushima.lg.jp/site/portal-english/ayumi-en-15.html). Eleven of the monitoring sites (nos. 1, 2, 7, 15, 23, 24, 25, 26, 27, 28, and 29) were located in the previously evacuated area. One of the monitoring sites (no. 26) remains located in the evacuated area.

### Sample collection for measurement of suspended radiocesium concentration and calculation of suspended radiocesium flux

#### Activity concentrations of suspended radiocesium *C*

Time-integrate SS samplers^31^ were set at 29 monitoring sites (i.e., all except for site no. 18). A bulk sample of SS was collected every few days to months. The samples were dried at 105‒110 °C for 24 hours. Then, the radiocesium concentration [*C* (Bq kg^−1^)] was measured using germanium semiconductor detectors (EGC25-195-R, GC3018, and GC4020 by Canberra-Eurisys, GEM20-70, GEM40P4-76, and GMX30-70-HJ by Ortec). All the data were corrected to the sample collection date.

Grain size distributions were also measured using laser diffraction/scattering particle size distribution analyzers (SALD-3100 by Shimazu, LA-960A by HORIBA) to obtain the mean diameter and grain size distribution of the sample. Since the laser-diffraction (LD) method can measure a wide range of particle size distributions, from sand to clay, the LD method has been widely used for particle size correction of Fukushima sediments containing a large amount of silt and clay. The specific surface area SSA [m^2^ g^−1^] was estimated using the grain size distribution and the spherical approximation of particles in each size fractions as per the following equation^9^.

1 $$SSA=\sum _{i}6\,{\rho }^{-1}{d}_{i}^{-1}{p}_{i},$$

where *p*_*i*_ and *d*_*i*_ [m] are the ratio and diameter of the grain size fraction, *ρ* is the particle density (i.e., 2.6 × 10^3^ kg m^−3^ for silica-sand).

The radiocesium concentrations for the period from August 2011 to January 2015 were previously published on the JAEA’s database site^25^. However, for the 29 samples collected between August 2011 and March 2012 at 10 sites (nos. 7–16), the data were measured without drying the samples. Also, all data were rounded to two significant digits. We obtained 12 samples of the 29 samples, dried them as well as the other samples for 24 hours at 105 °C, and then re-measured the radiocesium concentrations. The re-measured data, along with other initial data, were published again on the CRiED Database site^26^.

**Fig. 2:**
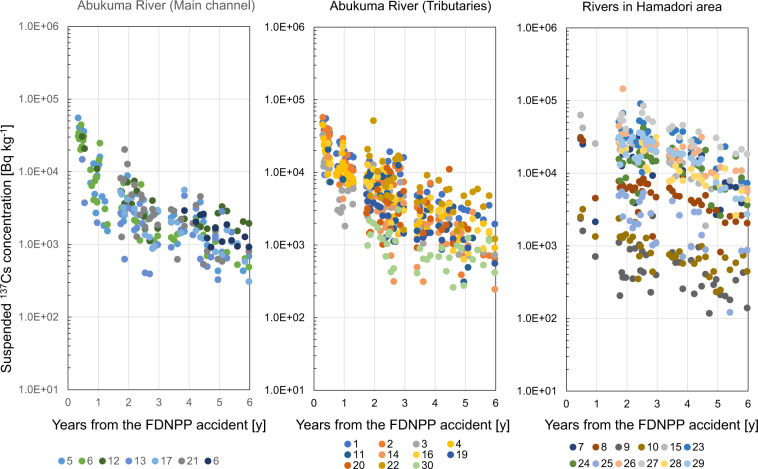
Time series of the radioactivity concentrations of suspended ^137^Cs. There are two types of points related to site no.6 in the left plot. The green point indicates the suspended ^137^Cs concentration collected on the left bank side and the dark blue point indicates the concentration collected on the right bank side. The points at the right bank were added in 2014 to confirm the representativeness of the data.

The time series of ^137^Cs radioactivity concentrations between June 2011 and March 2017 is shown in Fig. 2.

#### Radiocesium flux *L*

The suspended radiocesium (^134^Cs and ^137^Cs) fluxes (*L* [Bq month^−1^]) were obtained by the accumulation of the product of the activity concentrations of suspended radiocesium *C*, water discharge *Q* and suspended solid concentration *SSC* for one month as following equation.

2 $$L=\sum (Q\times SSC\times C)$$

**Fig. 3:**
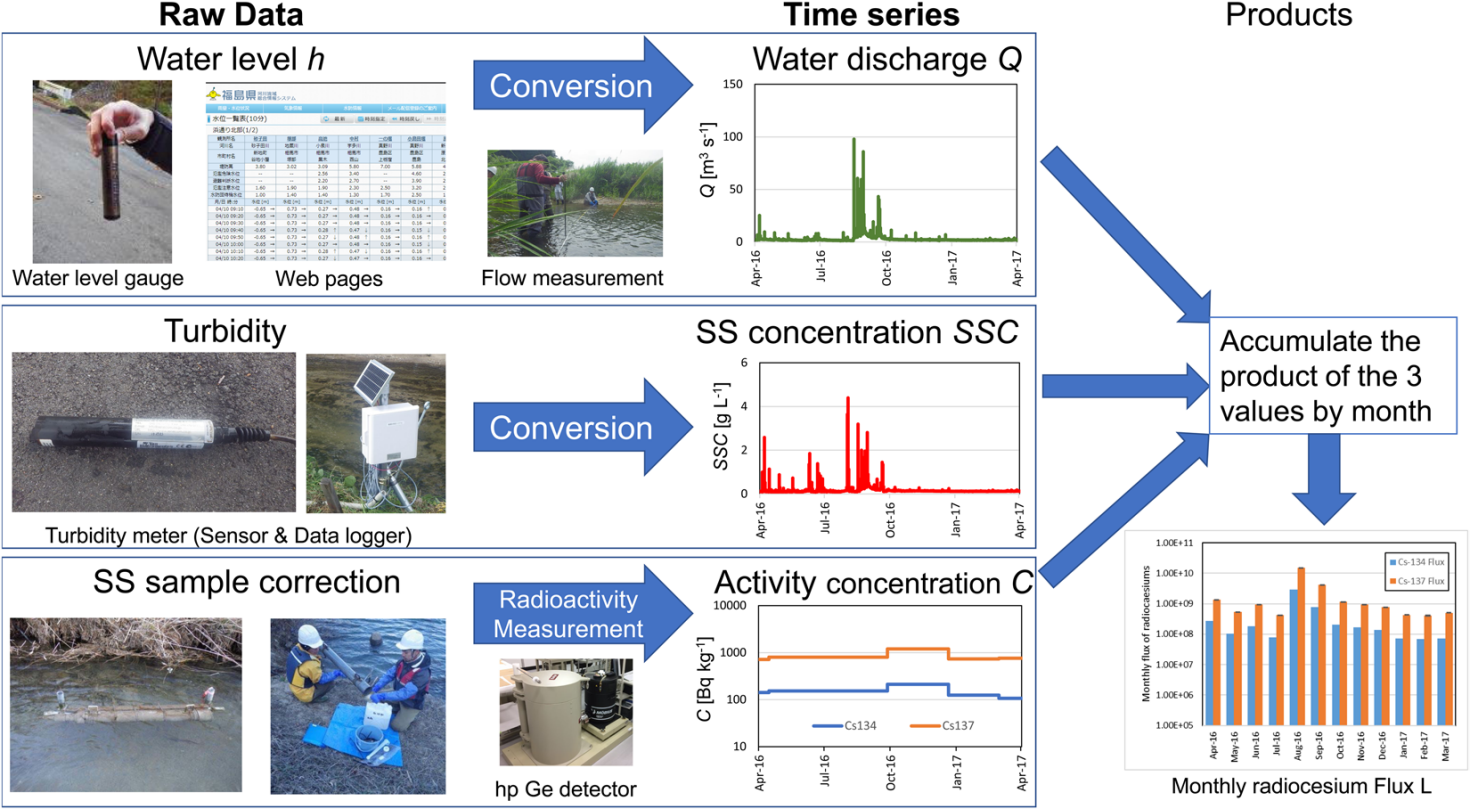
Schematic image of calculation of riverine radiocesium flux. The monthly fluxes of ^134^Cs and ^137^Cs were calculated by accumulating the water discharge (*Q*), SS concentration (*SSC*), and activity concentration (*C*) by month. The water discharge (*Q*) was converted from the water level (*h*) obtained from water level gauges or from the webpages of Ministry of Land, Infrastructure, Transport, and Tourism (MLIT) and Fukushima Prefecture using h-Q equations. The SS concentration was calculated from the turbidity using the conversion formula prepared using a standard solution. The activity concentrations of suspended ^134^Cs and ^137^Cs were obtained by measuring SS samples collected by SS samplers, dried at 105‒110 °C, and then measured with a hp Germanium semiconductor detector.

Figure 3 schematically illustrates the process of data collection and calculation.

The water discharge (*Q* [m^3^ s^−1^]) was calculated from the water level (*h* [m]). The water level data were obtained every 10 minutes from the web pages for 24 monitoring sites located near water level monitoring stations operated by Fukushima Prefecture (http://kaseninf.pref.fukushima.jp/gis/) and the Ministry of Land, Infrastructure, Transport, and Tourism (MLIT) (http://www1.river.go.jp/). At other sites (nos. 1, 2, 3, 7, 15, 26), we measured the water level every 10 minutes using a pressure type water level gauge (Rugged Troll 100 by In situ inc.). That water level data was converted to a flow rate (*Q*) using the h-Q equation. Equations for each of the monitoring sites were obtained from flow measurement. For 16 (nos. 5, 6, 9, 10, 12, 13, 14, 17, 19, 20, 21, 22, 24, 27, 29, 30) sites, we use the equations obtained by Fukushima Prefecture and MLIT, while equations for the other 13 sites (nos. 1, 2, 3, 4, 7, 8, 11, 15, 16, 23, 25, 26, 28) were made by the authors based on the data obtained by Fukushima Prefectural Centre for Environmental Creation. When long-term data loss occurred due to water level gauge loss, the flow rate estimated from the ratio with another monitoring station on the same or adjacent river was used.

The suspended solid concentration *SSC* was calculated from the turbidity data obtained every 10 minutes for 29 sites (i.e., all except for site 18). Initially, turbidity meters (NEP9350 by McVAN inc.) were installed at all sites. The turbidity meters were replaced with three different models after the 2015 fiscal year (SE-TV30MS by Senecom, Sensor DAKUDO by FieldPro, and ACLW2-USB by JFE Advantech) depending on the size of the river. All turbidity meters were calibrated using the same standard (bottom sediments in the Horai Dam reservoir on the Abukuma River). Then, conversion equations from turbidity to SSC were developed for all turbidity meters.

Turbidity data may remain unusable for long periods of time due to sensor failure, burial, or obstruction. In such cases, the data were complemented in the following two methods.

The power equation (Eq. 3) was used to estimate the monthly *Qss* (i.e. the products of *Q* and *SSC*) from the monthly flow rate *Q* for the data up to August 2015^10^:

3 $$({\rm{Monthly}}\,Qss)={\rm{a}}\times {\left({\rm{monthly}}Q\right)}^{{\rm{b}}},$$

where a and b are constants calculated from the monthly *Qss* and *Q*. In this case, the *L* is calculated as the product of *C* and monthly *Qss*.

For data since September 2015, *SSC*s were estimated from *Q* every 10 minutes using the linear rating curve shown below^28^:

4 $$SSC={\rm{c}}\times Q+{\rm{d}},$$

where c and d are constants calculated from the relationship between the *Q* and the *SSC* at the same point that was used to supplement the *SSC* value.

**Fig. 4:**
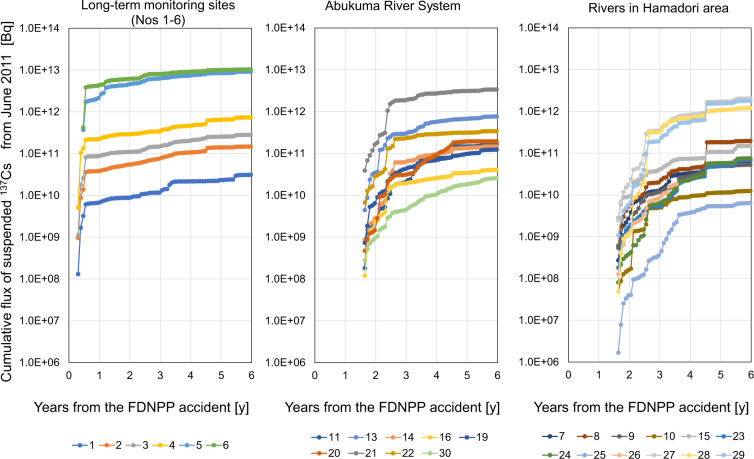
Cumulative flux of suspended ^137^Cs in the rivers in Fukushima area. The graph on the left shows the total fluxes since June 2011 for the six sites (nos. 1‒6) where turbidity has been observed since 2011. The graphs in the center and on the right show the accumulated fluxes since October 2012 for the other sites (nos. 7–11, 13–16, 19–30).

Figure 4 shows the monthly flux of suspended ^137^Cs accumulated from the start of the observation.

## Data Records

Data on the ^134^Cs and ^137^Cs concentrations and grain size distribution of SS between June 2011 and March 2017 and fluxes of suspended radiocesium from September 2015 to March 2017 is available on *Environmental Radioactivity Datasets website at CRiED, Univ. of Tsukuba* (http://www.ied.tsukuba.ac.jp/database/)^26–29^

Monthly ^137^Cs flux data from June 2011 to August 2015 were published in the supporting information of Taniguchi *et al*.^10^ (10.1021/acs.est.9b02890).

Table 2 summarized the number of data and differences in data processing methods between the two data sources.

**Table 2:** Data sources.

No.	1	2
**Source**	Supporting Information of Taniguchi *et al*.^10^ (https://pubs.acs.org/doi/10.1021/acs.est.9b02890)	Environmental Radioactivity Datasets website at CRiED, Univ. of Tsukuba (http://www.ied.tsukuba.ac.jp/database/)
Target	Flux of suspended ^137^Cs	Activity concentrations of suspended ^134^Cs and ^137^Cs Grain size distribution of the SS samples Flux of suspended ^134^Cs and ^137^Cs
Data number	27 Sites Total 1041 data	29 Sites Total 1917 data
Temporal range	Jun.2011 - Aug. 2015	Jun. 2011 - Mar. 2017 for radiocesium concentration Sep. 2015 – Mar. 2017 for the fiux of suspended ^134^Cs and ^137^Cs
Differences in calculation methods for flux	A power function was used to fill in the missing values for SSC.	A linear rating curve is used to fill missing SSC values
Files	es9b02890_si_001.pdf	DOI00014_data.csv DOI00015_data.csv DOI00020_data.csv DOI00021_data.csv

This dataset is available on two websites. Differences in data processing methods are also summarized.

## Technical Validation

### Uncertainty of radiocesium concentrations

The SS sampler, which was introduced by Phillips *et al*.^31^, has been reported as being problematic because it does not reproduce the particle size distribution of the suspended sand sized particles correctly due to the poor collection efficiency of fine particles^32^. As a result of water flume experiments, it was found that the collection efficiency improved as the flow velocity increased and the collection efficiency of particles with a diameter of <4 microns decreased^33^. This means that the SS sampler is able to collect SSs more efficiently during floods due to the higher flow velocity and larger mean grain size.

The adsorption capacity of cesium (including ^134^Cs and ^137^Cs) on SSs is dependent on the particle size^34,35^. Since the adsorption of cesium occurs at the surface, the radiocesium concentration of each size fractions can be represented by the power function of SSA^36^. In the case of rivers in Fukushima, the following equations can be used to correct for the effect of particle size distribution^9,37^:

5 $$C{\prime} =C/P,$$

and

6 $$P={\left({S}_{r}/{S}_{s}\right)}^{0.65}\,,$$

where *C* [Bq kg^−1^] and *C’* [Bq kg^−1^] are the measured and corrected values of radiocesium concentration, respectively; *P* is a particle-size correction factor; *S*_*r*_ and *S*_*s*_ are the SSA of the standard sample and measured sample.

The clay fraction by the LD method can be underestimated relative to that by the sieving/settling and pipette method in soil and coastal sediment cases^38,39^. Errors in the SSA caused by the difference in the measurement methods could not be quantitatively evaluated. A solution would be to avoid mixing data from different measurement methods in the cases of clay-rich sediments.

In the measurement of the radioactivity concentration by the Germanium semiconductor detector, only the counting errors were described. If the amount of sampled SS is small, the error is large. Although many types of detectors were used, the accuracy was checked by measuring standard samples to ensure that there were no differences in measurement results between detectors.

### Uncertainty of radiocesium fluxes

Errors in the ^134^Cs and ^137^Cs flux were assessed for data after September 2015^28^. The errors included in the three quantities (the activity concentration of radiocesium [*C*], water discharge [*Q*], and SS concentration [*SSC*]) used to calculate the flux are treated as follows:

1. The error in the radiocesium concentration *C* was considered only for the coefficient error of the measurement with the germanium semiconductor detector (*e*_*C*_).
2. Errors included in the water discharge *Q* were not evaluated. This is because the *h-Q* formula provided by the Ministry of Land, Infrastructure, Transport and Tourism (MLIT), and the Public Works Department of Fukushima Prefecture does not include information on the errors. However, these public agencies conduct surveys and data analysis in accordance with *Technical Guidelines for River Erosion Control* to maintain the quality of the *h*-*Q* formula (http://www.mlit.go.jp/river/shishin_guideline/index.html).
3. The error in the conversion from turbidity to *SSC* was not evaluated because it was confirmed to be highly linear (R^2^ > 0.99). When the *SSC* was complemented by the rating curve, the 95% prediction interval was used as the error *e*_*ssc*_.

From the law of propagation of error, the error *e*_*l*_ contained in the flux every 10 minutes is calculated as follows.

7 $$\begin{array}{l}l\pm {e}_{l}=Q\times \left(SSC\pm {e}_{SSC}\right)\times \left(C\pm {e}_{c}\right)\\ \quad \,=\left(Q\times SSC\times C\right)\pm \sqrt{{\left(C\times {e}_{SSC}\right)}^{2}+{(SSC\times {e}_{c})}^{2}}\end{array}$$

The error *e*_*L*_ included in the monthly flux *L* is expressed as follows.

8 $$L\pm {e}_{L}=\sum (l\pm {e}_{l})=\sum l\pm \sqrt{\sum {e}_{l}^{2}}$$
